# Variations in Presentation of Basilar Dolichoectasia: A Case Series

**DOI:** 10.7759/cureus.89537

**Published:** 2025-08-07

**Authors:** Manmeet Kaur, Aabhishkar Bhattarai, Ian A Orantes-Orellana, Yasamin Rastgar, Alok Dabi

**Affiliations:** 1 Neurology, University of Texas Medical Branch, Galveston, USA; 2 Medical School, University of Texas Medical Branch, Galveston, USA

**Keywords:** basilar dolichoectasia, hemi-facial spasms, hemorrhage, ischemic stroke, subocclusive thrombus

## Abstract

Vertebrobasilar dolichoectasia (VBD) is a vascular anomaly marked by abnormal elongation and dilation of the vertebral and basilar arteries. Often, VBD remains undiagnosed or is discovered incidentally during evaluations of vascular events such as ischemia, hemorrhage, hydrocephalus, or cranial nerve palsies. While most patients are managed conservatively, treatment choices are highly individualized based on clinical presentation, vessel characteristics, and risk factors. We report two cases of VBD identified incidentally following an ischemic stroke and hemifacial spasms. Although the cases share a similar underlying pathology, they exhibit entirely different manifestations, resulting in varied management strategies.

## Introduction

Vertebrobasilar dolichoectasia (VBD) is an uncommon but clinically significant vasculopathy characterized by elongation, dilation, and tortuosity of the vertebrobasilar artery. To be classified as dolichoectasia in the vertebrobasilar system, the basilar arterial diameter should be >4.5 mm. Also, Smoker's criteria are used to diagnose dolichoectasia, which uses three quantitative measures, including diameter, laterality, and height of bifurcation. VBD can be associated with chronic hypertension and aging and can present with multiple neurologic syndromes like ischemic strokes, transient ischemic attacks (TIAs), cranial nerve palsies, hydrocephalus, and sudden death due to brainstem compression [1-3].

VBD was first described in the early 20th century. Its prevalence in the general population ranges from 0.05% to 5%, with higher incidence reported in patients undergoing neuroimaging for cerebrovascular events [4]. Often diagnosed incidentally, VBD can lead to serious complications such as recurrent posterior circulation strokes with progressive neurologic disability. Imaging modalities such as magnetic resonance imaging (MRI) and magnetic resonance (MR) angiography are instrumental in diagnosis, demonstrating a dilated basilar artery with an elongated, tortuous course [5]. 

Management of VBD is challenging due to its rarity, the heterogeneity of clinical presentations, and the absence of a standardized treatment protocol. The current approach is conservative, focusing on vascular risk factor control, although some patients may require surgical or endovascular interventions for severe mass effect or hydrocephalus [6].

In this case series, we present two patients diagnosed with VBD, each with distinct clinical presentations and radiologic features. The objective is to improve awareness about the diagnostic complexity and therapeutic considerations in managing this challenging condition.

## Case presentation

Case report 1

A 64-year-old Caucasian male with a history of primary hypertension, hyperlipidemia, atrial fibrillation (managed with apixaban), and a prior right parieto-occipital stroke with residual mild left facial droop presented to the emergency department within an hour of new-onset vertigo, slurred speech, right-sided numbness, and mild weakness. The patient reported missing four consecutive doses of apixaban due to cost-related nonadherence. On initial evaluation, the National Institutes of Health Stroke Scale (NIHSS) score was three, reflecting left partial hemianopia, right upper limb drift, and chronic left facial weakness. Notably, the patient exhibited spontaneous clinical improvement within 5-10 minutes of presentation.

**Figure 1:**
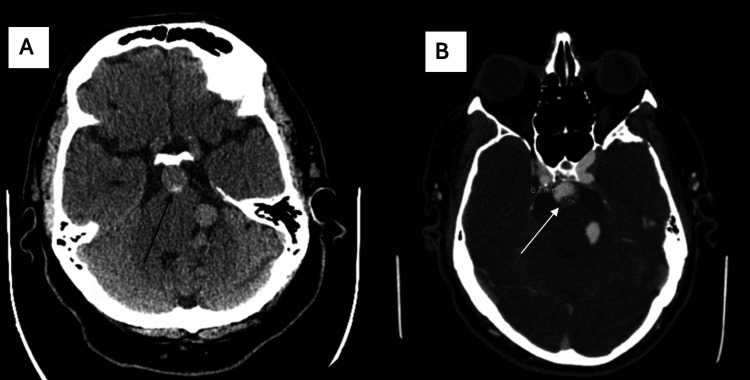
A. CT head without contrast, dolichoectatic vertebral artery. B. CTA head shows vertebrobasilar dolichoectasia noted with a large, long filling defect (thrombus) seen involving the mid basilar artery.

A non-contrast CT head revealed marked dolichoectasia of the left vertebral and basilar arteries with associated mass effect on the brainstem (Figure 1). CT angiography (CTA) could not be performed initially due to a reported history of contrast-induced anaphylaxis. The patient was pre-medicated with intravenous steroids and diphenhydramine in anticipation of delayed contrast imaging. Given the limited imaging and concern for a dissecting basilar artery aneurysm based on CT findings, dual antiplatelet therapy (DAPT) was empirically initiated, along with strict systolic blood pressure control targeting 120-160 mm Hg to mitigate the risk of further vessel compromise or rupture. Thrombolysis with tenecteplase was not administered due to a prior MRI-confirmed infarct within the last three months and the patient’s improving neurological symptoms.

The patient and family were unaware of any prior vascular abnormalities, and no prior vascular imaging was available from outside hospitals at the time of admission. However, within an hour of presentation, the patient's NIHSS score worsened to 15, with deficits including minor facial palsy (1), right arm paralysis (4), right leg weakness (3), bilateral limb ataxia (2), mild sensory loss (1), severe aphasia (2), and severe dysarthria (2). At that time, an urgent CTA of the head and neck was performed, which revealed vertebrobasilar dolichoectasia with a long, non-occlusive filling defect (thrombus) in the mid-basilar artery, causing approximately 50% luminal stenosis (Figure 1). Anticoagulation with intravenous heparin was initiated, and the patient’s NIHSS score improved to 11 by hospital day one.

**Figure 2:**
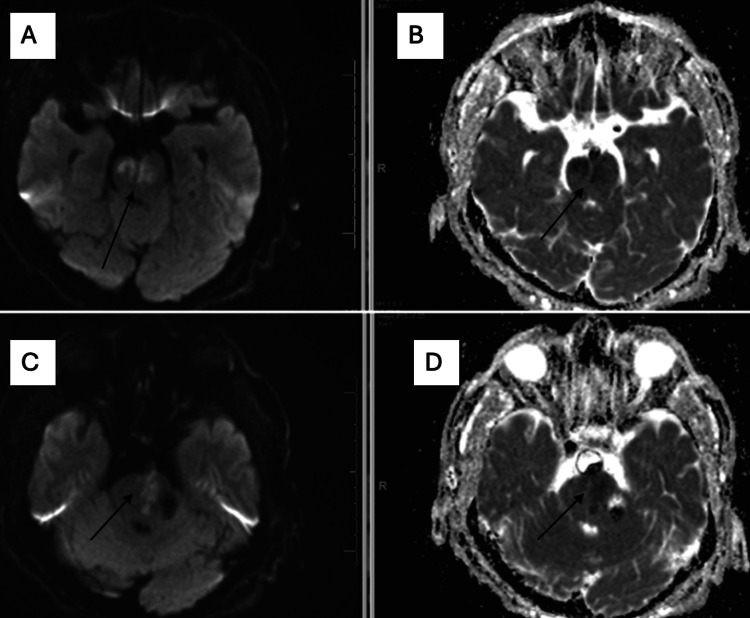
A and C: MRI of the brain without contrast demonstrates large areas of restricted diffusion within the pons, more prominent on the left side. B and C: Corresponding apparent diffusion coefficient (ADC) images show hypointensity in the same regions, consistent with acute infarction.

A digital subtraction angiogram (DSA) performed eight hours later confirmed a large, fusiform, dolichoectatic vertebrobasilar aneurysm with an intramural thrombus, 50% stenosis, and diffuse intracranial arterial dilatation. MRI of the brain showed asymmetric bilateral pontine infarctions and significant brainstem compression due to the dolichoectatic vertebrobasilar system (Figure 2). A transthoracic echocardiogram showed a mildly reduced ejection fraction (45-50%) with a moderately enlarged left atrium but no intracardiac thrombus or shunt.

The patient remained clinically stable during the remainder of his hospital stay. He was discharged to inpatient rehabilitation on day 10 with an NIHSS score of 8 and a modified Rankin Scale (mRS) score of 4. At a two-month follow-up in the stroke clinic, he demonstrated mild improvement in lower extremity strength but remained wheelchair-bound and dependent for most activities of daily living (ADLs). Tragically, 11 months after discharge, the patient suffered a catastrophic subarachnoid hemorrhage due to rupture of the tip of the dolichoectatic basilar artery aneurysm, resulting in his death.

Case report 2

**Figure 3:**
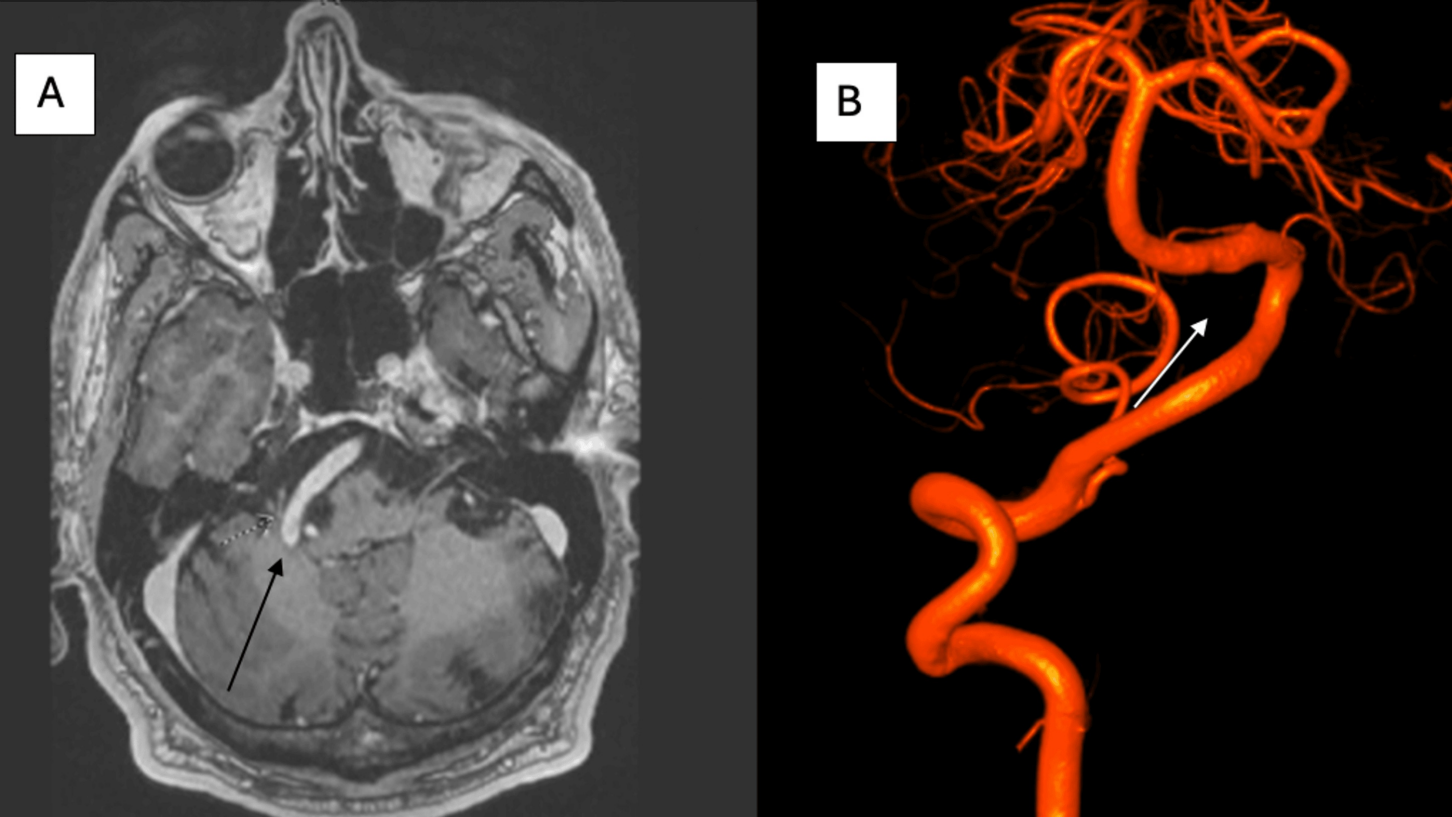
A. MRI of the brain with contrast showed dolichoectasia of the right vertebral artery, which crosses the midline to the left cerebellopontine angle and appears to exert a mass effect on the root entry zones of the left facial and vestibulocochlear nerves. B: Digital subtraction angiogram 3D construction showing tortuous elongated right vertebral with mild irregularities in lumen.

A 59-year-old male with a history of primary hypertension, hyperlipidemia, and a remote left pontine stroke two years prior presented to the neurology clinic with complaints of progressive left facial twitching and episodic vertigo, both of which had been present since 2017. He denied any residual motor or sensory deficits from the previous stroke, though neurological examination revealed a mild sensory deficit on the left side of the face in V2-V3 distribution. MRI of the brain with contrast demonstrated dolichoectasia of the right vertebral artery, which was seen crossing midline to the left cerebellopontine angle, exerting mass effect on the root entry zones of cranial nerves VII and VIII (Figure 3). Imaging also revealed a remote lacunar infarct in the pons.

This case represents one of the benign, slowly progressive presentations of vertebrobasilar dolichoectasia (VBD), as discussed in the introduction. While the chronic pontine infarct may be partially attributed to accelerated atherosclerotic changes in the vertebral artery due to dolichoectasia, there is no definitive evidence to establish this as the only cause. Notably, the patient was later diagnosed with paroxysmal atrial fibrillation on extended cardiac telemetry monitoring, which is a well-known independent risk factor for embolic stroke.

The patient’s hemifacial spasm was attributed to direct compression of the facial nerve by the dolichoectatic vertebral artery. He opted for conservative medical management. He was started on apixaban for secondary stroke prevention based on his elevated CHA₂DS₂-VASc score. Throughout the follow-up at 3 and 6 months, his facial twitching remained stable in terms of frequency and severity, and he experienced no new neurological events.

## Discussion

VBD refers to abnormal dilatation and elongation of the basilar artery. It is often associated with a variety of neurological symptoms and vascular complications, and its pathogenesis is complex, involving a combination of genetic, environmental, and vascular factors. One of the postulated hypotheses is the imbalance between extracellular matrix components, including collagen and elastin, and matrix metalloproteinases, which weaken the arterial wall, contributing to vessel dilatation and tortuosity [7-9]. Chronic systemic high blood pressure and atherosclerosis increase mechanical stress on vessel walls and may lead to arterial dilation [10]. The increased diameter and tortuosity of the artery might cause altered hemodynamics, including turbulent blood flow, that exacerbates vascular wall stress and contributes to vasodilatation [11]. Studies suggest that bidirectional endoscopy (BDE) may have a genetic component, especially in connective tissue disorders such as Ehlers-Danlos or Marfan syndrome. Mutations in collagen synthesis and connective tissue integrity genes, such as COL3A1 and FBN1, may increase the risk for BDE. Chronic low-grade inflammation may also contribute to vascular remodeling and endothelial dysfunction [9].

In case 1, the patient's stroke was likely caused by thrombus formation within the dolichoectatic basilar artery. The rapid clinical deterioration, combined with CTA and DSA findings of a non-occlusive thrombus and fusiform dilation, supports a thromboembolic mechanism. Although the patient was also in atrial fibrillation and had missed doses of apixaban, it is probable that the altered flow dynamics within the ectatic vessel contributed directly to thrombus development. The contribution of ectasia in such cases is not incidental but pathogenic.

In case 2, the patient exhibited one of the more benign manifestations of VBD with progressive hemifacial spasm due to direct compression of the facial nerve. MRI showed the dolichoectatic vertebral artery displacing cranial nerves VII and VIII at the root entry zone. Although a chronic pontine infarct was also seen, its etiology remains uncertain and possibly related to chronic vertebrobasilar hypoperfusion or paroxysmal atrial fibrillation diagnosed later. Nonetheless, this case emphasizes that VBD can present insidiously and may remain clinically stable for years.

Vertebrobasilar dolichoectasia significantly increases the risk of ischemic and hemorrhagic strokes, dissection, and arterial rupture [2]. Many patients are asymptomatic and diagnosed incidentally. Symptoms result from compression of adjacent structures by the abnormal vasculature, thromboembolism, or altered blood flow [12,13]. In our first case, a thrombus in the dolichoectatic basilar artery led to pontine infarction. This is a serious complication, with brainstem ischemia and potential rapid clinical deterioration [14]. Compression of cranial nerves and brainstem structures may lead to cranial neuralgias, ataxia, bulbar symptoms, or hydrocephalus [6,13]. Catastrophic complications such as aneurysmal rupture or acute thrombotic occlusion are not uncommon. 

Given the variability in presentation, management must be individualized. Conservative management, including risk factor modification and medical therapy (e.g., antiplatelets, anticoagulants), is often appropriate for asymptomatic or mildly symptomatic patients [10,11]. In selected cases, endovascular or surgical interventions may be required to address thrombosis, aneurysm, or significant mass effect.

## Conclusions

Basilar dolichoectasia is a rare but clinically important vascular anomaly with variable clinical presentation, ranging from an incidental imaging finding to life-threatening brainstem ischemia and compression. The cases presented here underscore the heterogeneity of BDE’s clinical manifestations and the importance of a thorough diagnostic workup and maintaining a high index of suspicion. Early recognition through appropriate imaging modalities, with aggressive risk factor management, helps optimize outcomes. Management is often conservative, and individualized therapeutic strategies, like anticoagulation, antiplatelet therapy, or surgical intervention, may be necessary based on specific risk factors, like thromboembolic risk, mass effect, or progressive neurological deficits. In summary, these cases help raise awareness of BDE’s natural history and emphasize the need for an individualized, multidisciplinary approach in managing this complex cerebrovascular condition. Future longitudinal studies are warranted to better define optimal surveillance strategies and intervention thresholds for asymptomatic and symptomatic patients.
